# Supplementary material for: What influences uptake and early adherence to Option B+ (lifelong antiretroviral therapy among HIV positive pregnant and breastfeeding women) in Central Uganda? A mixed methods study
Source: PLoS One. 2021 May 5;16(5):e0251181. doi: 10.1371/journal.pone.0251181 (PMC8099116; doi:10.1371/journal.pone.0251181)
Supplement: S1 Appendix — (PDF) [file pone.0251181.s001.pdf]

S1 Appendix: Key Informant interview Guide

Implementation of Option B+ for PMTCT in Uganda: Assessing the uptake and retention of mothers and their infants into PMTCT services.

Position of interviewee in clinic: ________________________

Cadre of interviewee: ________________________________

Number of years worked with HIV: _____________________

Interviewer: _______________________________________

Date of interview: ___________________________________

Location of interview/clinic name: _______________________

Duration of interview: Time Started: |__|__| Hour |__|__| Minutes
Time Ended: |__|__| Hour |__|__| Minutes

Implementation of Option B+ for PMTCT in Uganda: Assessing the uptake and retention of mothers and their infants into PMTCT services.

About this study

This research study is being conducted by Makerere University School of Public Health with support from the Ministry of Health. The purpose of this research is to gather information mainly on acceptance (uptake) of lifelong antiretroviral therapy (ART) among HIV infected pregnant women, retention in care for the women and their infants; and adherence to ART to help strengthen the PMTCT services across the country. This study also seeks to examine provider experiences and support mechanisms for clients to ensure retention and adherence to ART as well as challenges and successes in the PMTCT programs in relation to delivery of ART within the selected facilities in Masaka, Luwero and Mityana districts.

We are asking you to participate in this study because you are a health worker (or expert client) in this health facility which is one of the facilities selected to participate in the study.

1. Describe your roles or responsibilities in PMTCT service delivery in this health facility?

Now, I would like to ask you questions on how PMTCT services are delivered in this facility.

2. What information do you give the pregnant HIV+ women about Option B+?
   a. In your experience, how sufficient is the information you give to the women? What are the gaps? Probe:
      - When to start lifelong ART/who should start?
      - What happens after giving birth?
      - What happens after breastfeeding?

   b. Who does the prescriptions for lifelong ART among pregnant and breastfeeding women, and monitoring of treatment outcomes including side-effects, and where are these done? Probe:
      - Prescription, monitoring, cadre that does these.
      - Where are these done (referrals or within this facility)

3. Please tell me what you understand as the benefits of option B+? Probe: benefits to the mother, child, spouse

4. What challenges do you face in supporting women on option B+? Probe:
   - Support for retention, adherence.
   - Concerns about drug resistance and how to avoid it.
   - Drug stock outs, staffing numbers and trainings/skills.

5. In your experience, what challenges do pregnant women on Option B+ face? Probe:
    - Loss to follow up, adherence, drug stock outs.
    - Stigma, discrimination
    - Referrals across difference service points such as ANC, ART.

Now, I would like you to tell me about the process of preparing for the initiation and support of pregnant women for Option B+ in this facility.

6. In your view, how acceptable is option B+ to the women? What concerns do women raise? What happens if a woman refuses to take the Option B+ drugs?

7. How do you prepare the women before initiating treatment? Probe:
    - Counseling and other processes; for how long,
    - Understanding of adherence and retention requirements,
    - Need for treatment supporter,
    - Testing and involvement of the husband,

Now, I would like you to tell me about the strategies used in this facility to ensure mothers remain in care.

8. When do you say that a mother is lost to follow-up in this facility? What factors do you consider to be the main contributors to loss to follow-up? Probe: Does retention vary across pregnancy, child birth and breastfeeding?

9. What retention support strategies are being used? Probe: Family support group, mentor mothers, telephone reminders.

10. How do you support the women after initiating Option B+? Probe: Procedures for detecting and tracking women who miss appointments; male involvement; detecting and tracking the exposed child.

11. To what extent does stigma affect adherence?

12. What measures has the clinic instituted to address the challenges related to stigma? Probe: How effective are these strategies in helping women remain in care?

Now, I would like you to tell me about adherence to HAART for pregnant women/Option B+.

13. How do you detect women who have adherence problems? What factors do you consider to be the main contributors to non-adherence to HAART for pregnant women/Option B+? Probe: does adherence vary across pregnancy, child birth and breastfeeding?

14. What adherence support strategies are being used to support pregnant women on Option B+? How do you support the women after initiating Option B+?

    Probe:

    - Procedures for detecting and tracking women who are non-adherent
    - Male involvement;
    - Detecting and tracking the exposed child,
    - Treatment buddy
    - Family support group, mentor mothers, telephone reminders.

Now, I would like you to tell me about how clinics for women and children services are organized in this facility. (Interviewer: Ask these next 2 questions at the same time)

15. How are the clinics for women services organized? What linkages exist between ANC/PNC/PMTCT, EID and chronic care HIV clinics? What happens to the women who initiate Option B+ after delivery? Probes:
    - Do they continue to get drugs at the same site/clinic or do they transfer elsewhere?
    - Do they continue under the same providers? If they transfer, how is the transfer or referral done?
    - How soon after delivery do, they transfer? Are there any challenges in this transfer/referral process?
    - How do providers ascertain completion of the referral process?

16. Are there any other issues you would like to share with us?

Thank you very much for your time
