# Supplementary material for: What influences uptake and early adherence to Option B+ (lifelong antiretroviral therapy among HIV positive pregnant and breastfeeding women) in Central Uganda? A mixed methods study
Source: PLoS One. 2021 May 5;16(5):e0251181. doi: 10.1371/journal.pone.0251181 (PMC8099116; doi:10.1371/journal.pone.0251181)
Supplement: S2 Appendix — (PDF) [file pone.0251181.s002.pdf]

S2 Appendix: In-depth interview Guide

Implementation of option B+ for PMTCT in Uganda: Assessing the uptake and retention of mothers and their infants into PMTCT services

Age of the respondent: ________________________________

Education level: _____________________________________

Marital status: _______________________________________

Number of previous pregnancies: ________________________

Interviewer: ____

Date of interview: ____________________________________

Location of interview/clinic name: ________________________

Duration of interview: Time Started: |__|__| Hour |__|__| Minutes
Time Ended: |__|__| Hour |__|__| Minutes

Implementation of Option B+ (Lifelong ART) for PMTCT in Uganda: Assessing the uptake and retention of mothers and their infants into PMTCT services.

About this study

This research study is being conducted by Makerere University School of Public Health with support from the Ministry of Health. The purpose of this research is to gather information mainly on acceptance (uptake) of antiretroviral therapy (ART) among HIV infected pregnant women, retention in care for the women and their infants; and adherence to ART to help strengthen the PMTCT services across the country. This study also seeks to examine provider experiences and support mechanisms for clients to ensure retention and adherence to ART as well as challenges and successes in the PMTCT programs in relation to delivery of ART within the selected facilities in Masaka, Luwero and Mityana districts.

We are asking you to participate in this study because you have been receiving care from this health facility which is one of the facilities selected to participate in the study.

First, I would like you to tell me about your journey towards starting Option B+ ART

1. Describe your road to starting ART (Option B+). When and how did you decide to test for HIV? Probes:
    - Was it done at this ANC clinic?
    - Did you know your HIV positive status before attending the clinic?
    - What were you told about the ART?
    - How long were you told you will be taking the ART?

2. Once you tested HIV+, how did you come to be offered ART? What were you told about the lifelong ART (Option B+?) From who? Probes:
    - What were you told about the benefits of lifelong ART (Option B+) for your health, child and spouse?
    - What were you told about prevention of vertical transmission?
    - What were you told about treatment for yourself?
    - When to start ART/who should start; what happens after giving birth; what happens after breastfeeding?
    - Were you first provided with some pre-treatment education and orientation sessions before you started taking ART? If so, how many, and what did they consist of?
    - Did you find these sessions useful in helping you cope with taking the ART drugs?
    - Would you say the information you were given is sufficient? What are the gaps?

3. What do you understand as the benefits of Option B+?
    - What are the potential benefits of Option B+?
    - Does Option B+ provide any benefit to communities or partners of pregnant women*: opportunity for husbands to test by proxy, couples counseling, reducing risk of infection to husbands.*

5(a). Why and how did you make the decision to start Option B+? **(Interviewer: This question is applicable for mothers already on Option B+)**

- What motivated you to start? Probe: major reasons for starting Option B+?
- Did you have any concerns about starting option B+? **[Interviewer: Please probe, if necessary]**
- What are your opinions about Option B+?
- When were you asked / expected to start treatment? Was it the same day they found out your HIV status?
- Did you feel you were ready to start Option B+ ART at that point?

5(b). Why and how did you make the decision to decline starting Option B+? **(Interviewer: This question is applicable for mothers who declined to start Option B+)**

- What reasons did you have for declining to start Option B+?
- Did you have any concerns about starting option B+? **[Interviewer: Please probe, if necessary. E.g., willingness]**
- What are your opinions about Option B+?
- When were you asked / expected to start treatment? Was it the same day they found out your HIV status?

6. Have any women refused to begin treatment?
    - What is the response from the providers if a woman says she does not want to begin treatment?
    - What information do women need to make a decision about whether to begin treatment?
    - In what ways should women be given this information?

7. What challenges have you faced with Option B+? **(Interviewer: For women already on Option B+)**
    - What challenges have other women faced with Option B+?
    - What can be done to overcome these challenges? Probe: the women, family and facilities. Probes: *loss to follow up, adherence, drug stock outs.*

8. Option B+ is offered to the special population of pregnant women living with HIV but not all people living with HIV? What challenges might this cause?

9. People often find it difficult to closely follow ART dosing instructions and occasionally miss doses. Have you had any problems taking all of your medication doses? Probe:
   - Disclosure; husband involvement and support, working hours.
   - What have been the main reasons for missing the doses?

10. Have you had any challenges keeping all your clinic appointments? Probe:
    - Is transportation a problem related to making your clinic appointments? **[Interviewer: If the woman keeps all appointments; ask what has made her keep all the appointments and if the woman does not keep all appointments, ask what has made her fail to keep all these appointments]**

11. To what extent does stigma related to HIV affect the ability of women to take Option B+ in this community? What should be done to address this?

12. Is there anything else you would like to share with us?

Thank you for your time
