# Supplementary material for: What influences uptake and early adherence to Option B+ (lifelong antiretroviral therapy among HIV positive pregnant and breastfeeding women) in Central Uganda? A mixed methods study
Source: PLoS One. 2021 May 5;16(5):e0251181. doi: 10.1371/journal.pone.0251181 (PMC8099116; doi:10.1371/journal.pone.0251181)
Supplement: Attachment — Submitted filename: Response to Reviewers.docx [file pone.0251181.s005.docx]

PONE-D-20-22840R1

What influences uptake and early adherence to Option B+ (lifelong antiretroviral therapy among HIV positive pregnant and breastfeeding women) in Central Uganda? A mixed methods study

PLOS ONE

Dear Dr. Mukose,

Thank you for submitting your manuscript to PLOS ONE. After careful consideration, we feel that it has merit but does not fully meet PLOS ONE’s publication criteria as it currently stands. Therefore, we invite you to submit a revised version of the manuscript that addresses the points raised during the review process.

I have gone through the manuscript for final edits. Please review the version of the manuscript in which I have made changes and comments. Please add explanations as needed and send back your final edited version.

Response to comments:

Dear Reviewers and the Academic Editor, we thank you for your time to review our responses. We also thank you the Academic Editor for going through the manuscript for final edits.

We have added the explanations as needed. Please find detailed point by point explanations and the changes highlighted in the “Revised Manuscript with Track Changes”.

Dear the Academic Editor, please find detailed point by point explanations.

1. Not clear what these mean (HC IV, HC III). Are these designations necessary? Add explanation if needed, delete if not needed. Be consistent throughout and define all or delete all

Dear Editor, according to the structure of the Uganda public health-care system, there are seven levels of health care delivery, organized from lower to higher levels in a hierarchy. HC IV means health center 4 whereas HC III refers to health center 3. A reference (Mukose, Aggrey David, et al. "Health Provider Perspectives of Health Facility Preparedness and Organization in Implementation of Option B+ among Pregnant and Lactating Women in Central Uganda: A Qualitative Study." Journal of the International Association of Providers of AIDS Care (JIAPAC) 18 (2019): 2325958219833930) has been included. Please see lines 124-125 on page 7.

2. Explain the modifications. Not clear whether the references indicate the modified or unmodified questionnaire.

The references indicate the unmodified questionnaire. We picked questions and issues that were used in the references. They were used to come up with the final questionnaire that was used our study. The modifications ensured that the questionnaire was context specific.

3. A 5% precision was determined using the sample sizes that we used for the two study outcomes (levels of uptake, and early adherence). Unclear what this means. Please clarify

Dear Academic Editor, we have clarified the sentence to read as: Basing the sample sizes that we used for the two study outcomes, (levels of uptake; and early adherence), the study had a 5% level of precision. Lines 157-159, page 8.

4. Analysis revealed that there was no interaction.

Thank you so much Editor we have moved the sentence “Analysis revealed that there was no interaction” from the methods section (Line 240, page 12) to the results section; (line 510, page 32)

5. Relative risks are indicated, but no interactions were found among factors discussed below. This needs to be in the results somewhere. This is just a suggestion. Could add a small section at the end of the results instead (Page 13).

Thank you so much. This has been modified and added to the section on “Factors associated with optimal early adherence". Please see lines 509 -510 on page 32.

6. 05_IDI, 02_IDI etc.

“05_IDI” was used to link the quote to the in-depth interview participant. We have replaced it with “IDI Participant” throughout the manuscript.

7. Academic Editor: Maybe move this paragraph (Recommendations) to the end of the Implications section and delete the header.

Reviewer #1: The authors should place the recommendation section in the manuscript before

conclusion.

Thank you so much the Academic Editor and Reviewer #1. We have moved the section on recommendations from a separate section (Lines 674 -680 on page 42) to the end of the implications section and deleted the header. Please see lines 643-648, page 40.

Please submit your revised manuscript by May 15 2021 11:59PM. If you will need more time than this to complete your revisions, please reply to this message or contact the journal office at plosone@plos.org. When you're ready to submit your revision, log on to https://www.editorialmanager.com/pone/ and select the 'Submissions Needing Revision' folder to locate your manuscript file.

Please include the following items when submitting your revised manuscript:

A rebuttal letter that responds to each point raised by the academic editor and reviewer(s). You should upload this letter as a separate file labeled 'Response to Reviewers'.

A marked-up copy of your manuscript that highlights changes made to the original version. You should upload this as a separate file labeled 'Revised Manuscript with Track Changes'.

An unmarked version of your revised paper without tracked changes. You should upload this as a separate file labeled 'Manuscript'.

If you would like to make changes to your financial disclosure, please include your updated statement in your cover letter. Guidelines for resubmitting your figure files are available below the reviewer comments at the end of this letter.

If applicable, we recommend that you deposit your laboratory protocols in protocols.io to enhance the reproducibility of your results. Protocols.io assigns your protocol its own identifier (DOI) so that it can be cited independently in the future. For instructions see: http://journals.plos.org/plosone/s/submission-guidelines#loc-laboratory-protocols

We look forward to receiving your revised manuscript.

Kind regards,

Julie AE Nelson, PhD

Academic Editor

PLOS ONE

Journal Requirements:

Please review your reference list to ensure that it is complete and correct. If you have cited papers that have been retracted, please include the rationale for doing so in the manuscript text, or remove these references and replace them with relevant current references. Any changes to the reference list should be mentioned in the rebuttal letter that accompanies your revised manuscript. If you need to cite a retracted article, indicate the article’s retracted status in the References list and also include a citation and full reference for the retraction notice.

[Note: HTML markup is below. Please do not edit.].

Reviewers' comments:

Reviewer's Responses to Questions

Comments to the Author

1. If the authors have adequately addressed your comments raised in a previous round of review and you feel that this manuscript is now acceptable for publication, you may indicate that here to bypass the “Comments to the Author” section, enter your conflict of interest statement in the “Confidential to Editor” section, and submit your "Accept" recommendation.

Reviewer #1: All comments have been addressed

Reviewer #2: All comments have been addressed

2. Is the manuscript technically sound, and do the data support the conclusions?

The manuscript must describe a technically sound piece of scientific research with data that supports the conclusions. Experiments must have been conducted rigorously, with appropriate controls, replication, and sample sizes. The conclusions must be drawn appropriately based on the data presented.

Reviewer #1: Yes

Reviewer #2: Yes

3. Has the statistical analysis been performed appropriately and rigorously?

Reviewer #1: I Don't Know

Reviewer #2: Yes

4. Have the authors made all data underlying the findings in their manuscript fully available?

The PLOS Data policy requires authors to make all data underlying the findings described in their manuscript fully available without restriction, with rare exception (please refer to the Data Availability Statement in the manuscript PDF file). The data should be provided as part of the manuscript or its supporting information, or deposited to a public repository. For example, in addition to summary statistics, the data points behind means, medians and variance measures should be available. If there are restrictions on publicly sharing data—e.g. participant privacy or use of data from a third party—those must be specified.

Reviewer #1: Yes

Reviewer #2: Yes

5. Is the manuscript presented in an intelligible fashion and written in standard English?

PLOS ONE does not copyedit accepted manuscripts, so the language in submitted articles must be clear, correct, and unambiguous. Any typographical or grammatical errors should be corrected at revision, so please note any specific errors here.

Reviewer #1: Yes

Reviewer #2: Yes

6. Review Comments to the Author

Please use the space provided to explain your answers to the questions above. You may also include additional comments for the author, including concerns about dual publication, research ethics, or publication ethics. (Please upload your review as an attachment if it exceeds 20,000 characters)

Reviewer #1: The authors should place the recommendation section in the manuscript before conclusion. Thank you. I have no further comments.

Thank you so much the Academic Editor and Reviewer #1. We have moved the section on recommendations from a separate section (Lines 674 -680 on page 42) to the end of the implications section and deleted the header. Please see lines 643-648, page 40.

Reviewer #2: Thank you very much for taking time to address the review comments. The revised manuscript and revision comments are well received.

7. PLOS authors have the option to publish the peer review history of their article (what does this mean?). If published, this will include your full peer review and any attached files.

If you choose “no”, your identity will remain anonymous but your review may still be made public.

Do you want your identity to be public for this peer review? For information about this choice, including consent withdrawal, please see our Privacy Policy.

Reviewer #1: Yes: Dr Olumuyiwa Omonaiye

Reviewer #2: Yes: Ezekiel Mupere MBChB, MMed, MS., PhD (Epidemiology & Bio-statistics)

[NOTE: If reviewer comments were submitted as an attachment file, they will be attached to this email and accessible via the submission site. Please log into your account, locate the manuscript record, and check for the action link "View Attachments". If this link does not appear, there are no attachment files.]

While revising your submission, please upload your figure files to the Preflight Analysis and Conversion Engine (PACE) digital diagnostic tool, https://pacev2.apexcovantage.com/. PACE helps ensure that figures meet PLOS requirements. To use PACE, you must first register as a user. Registration is free. Then, login and navigate to the UPLOAD tab, where you will find detailed instructions on how to use the tool. If you encounter any issues or have any questions when using PACE, please email PLOS at figures@plos.org. Please note that Supporting Information files do not need this step.

Thank you so much Reviewers for accepting all our responses.
